# Improving engagement with healthcare in hepatitis C: a randomised controlled trial of a peer support intervention

**DOI:** 10.1186/s12916-019-1300-2

**Published:** 2019-04-01

**Authors:** Helen R. Stagg, Julian Surey, Marie Francis, Jennifer MacLellan, Graham R. Foster, André Charlett, Ibrahim Abubakar

**Affiliations:** 1 Institute for Global Health, University College London, 4th floor, Mortimer Market Centre, off Capper Street, London, WC1E 6JB UK; 2 Usher Institute of Population Health Sciences and Informatics, The University of Edinburgh, MacKenzie House, 30 West Richmond Street, Edinburgh, EH8 9DX UK; 3 Barts Liver Centre, Blizard Institute, Queen Mary University of London, London, E1 2AT UK; 4 Statistics, Modelling and Economics Department, Public Health England, 61 Colindale Avenue, London, NW9 5EQ UK

**Keywords:** Hepatitis C, Peer support, Peer advocacy, Peer worker

## Abstract

**Background:**

Peer support can enable patient engagement with healthcare services, particularly for marginalised populations. In this randomised controlled trial, the efficacy of a peer support intervention at promoting successful engagement with clinical services for chronic hepatitis C was assessed.

**Methods:**

In London, UK, potential participants were approached through outreach services for problematic drug use and homelessness. Individuals positive for hepatitis C virus (HCV) after confirmatory testing were randomised using an online service to the intervention (peer support) or standard of care. The primary outcome of interest was successful engagement with clinical hepatitis services. The study was non-blinded. Absolute differences were calculated using a generalised linear model and the results compared to logistic regression.

**Results:**

Three hundred sixty-four individuals consented to participate. One hundred one had chronic hepatitis C and were randomised, 63 to receive the intervention (peer support). A successful outcome was achieved by 23 individuals in this arm (36.5%) and seven (18.4%) receiving the standard of care, giving an absolute increase of 18.1% (95% confidence interval 1.0–35.2%, *p* value = 0.04). This was mirrored in the logistic regression (odds ratio 2.55 (0.97–6.70), *p* = 0.06). No serious adverse events were reported.

**Conclusions:**

Peer support can improve the engagement of patients with chronic HCV with healthcare services.

**Trial registration:**

ISRCTN24707359. Registered 19th October 2012.

**Electronic supplementary material:**

The online version of this article (10.1186/s12916-019-1300-2) contains supplementary material, which is available to authorized users.

## Background

In England, around 160,000 individuals were estimated to be chronically infected with hepatitis C virus (HCV) in 2017, a large proportion of whom are from underserved and marginalised groups [1]. The UK has committed to eliminating HCV by 2025 [2]; thus, it is critical that all population groups are able to access effective diagnostic and treatment services.

Underserved and vulnerable groups are defined by factors that render engagement with normal healthcare services and pathways problematic, which often results in them being labelled as ‘hard-to-reach’ by such systems. They include homeless persons, people who inject drugs and ex-prisoners. These groups can be vulnerable to a range of infectious diseases due to living conditions, exposure to injecting drug use, alcoholism, generally poor physical and mental health and socio-structural factors that criminalise, isolate and stigmatise. Exposure to, and prevalence of, hepatitis B virus (HBV) and HCV among marginalised groups in the UK is known to be high [3–5]. Whilst testing for HCV and HBV is not uncommonly offered by UK outreach services, this is not sufficient to promote effective engagement through the full diagnostic and treatment process.

The treatment landscape for chronic HCV has been revolutionised in the past decade, with the licencing of a series of directly acting antivirals (DAAs) for HCV [6]. Although expensive, these drugs have radically improved the patient treatment experience, shortened the duration of therapy, reduced the likelihood of adverse events and have better (pan-genotype) treatment success rates. Such regimens offer hope to all chronically infected individuals, but most especially those who may struggle to access specialist services and adhere to treatment. Monthly treatment targets of chronic HCV patients have been set for each Operational Delivery Network (ODN) in England; as hospitals work through their lists of patients awaiting DAAs, greater efforts will be needed to reach the individuals less likely to engage [7]. This includes those currently injecting drugs, a key population if elimination of HCV is to be achieved. Underserved and vulnerable groups as a whole will act as a reservoir of infection if their healthcare needs are not addressed.

Peer support is a mechanism to enable active engagement with healthcare among marginalised groups. Peers have personal experience of a specific illness or lifestyle that enables them to support others experiencing similar challenges [8]. Peer support has been used worldwide, particularly in mental health, to remove barriers to accessing services, facilitate patient wellbeing and promote positive clinical outcomes [8]. In chronic hepatitis C, peers have been documented as playing a wide range of roles [9–11]; helping individuals to engage with their treatment is a particularly critical area [12, 13]. Qualitative and observational studies have highlighted peer support models as valuable in the care of chronically infected HCV patients in Australia, Georgia, the UK and the USA [12–25], but quantitative trial data is lacking. Peer support has been deemed one of ten priorities to improve access to HCV treatment for people who inject drugs in low- and middle-income countries [26].

Given the promising observational data and qualitative evidence, and the absence of quantitative trial data on the value of peer support for promoting successful engagement with clinical services for chronic HCV, we conducted a randomised controlled trial (RCT) aimed to evaluate the efficacy of an individual-level peer support intervention.

## Methods

### Study design

This was a randomised non-blinded controlled trial in London, UK, to assess the efficacy of a community-controlled, individual-level, peer support intervention to promote engagement with healthcare services in individuals chronically infected with HCV.

### Participants

Potential participants were approached at outreach services for problematic drug use and homelessness for point-of-care HCV, HBV, and HIV testing. Inclusion criteria were being marginalised by normal healthcare services (evidenced by engagement with outreach services as a client), over the age of 16 years, and willing and able to provide written informed consent. Potential participants were excluded if they were already on treatment for HCV or HBV. Additionally, individuals known by outreach services to be positive for HCV and/or HBV who were not on treatment (‘known positives’) were approached.

### Testing procedures

All consenting participants were asked to complete a baseline questionnaire (Additional file 1) by the study Research Nurses, which collected self-reported demographic, medical and contact information. They were offered point-of-care testing for HCV (OraQuick anti-HCV), HBV (nal von Minden HBsAg) and HIV (Alere third generation). Participants could choose to have less than the full complement of tests.

Participants found to be point-of-care test positive for HCV or HBV were informed at the time of screening and had venous and/or dried blood spot samples of blood taken for confirmatory testing by Public Health England (PHE), which provides a national reference service for HCV and HBV testing. Confirmatory antibody testing for chronic infection was undertaken for HCV and antigen/antibody testing for HBV; if positive, this was followed by polymerase chain reaction (PCR) testing for genetic material and genotyping. Individuals positive for HIV at the point-of-care testing stage were informed and the results provided to their primary care practitioner, if permission was given.

Anyone testing positive for HCV or HBV at this confirmatory stage was fully enrolled into the trial and randomised to the intervention or standard care arms.

### Randomisation and masking

Randomisation was undertaken centrally by the research nurses using the web-based Sealed Envelope system. Eligible enrolled participants were randomised 1:2 standard of care to intervention arm. Additional details are presented in Additional file 2, including the sample size calculation.

### Standard of care

Enrolled participants not randomised to the intervention arm were referred to one of four hospitals (The Royal London/Barts Health, King’s College London, Royal Free, University College). Their test results—and notification of their study participation—were sent to their primary care practitioner, if permission was given. Individuals were allowed to choose which hospital to be referred to, regardless of their study arm. There was no further intervention by the trial team. The specific site of referral for both arms of the study was chosen based on participant preference and geographical location prior to randomisation results being known. Referral sites did not explicitly know the randomisation status of the patients.

### Intervention

In our community-controlled model of peer support, participants in the intervention arm were individually assigned to a peer advocate from the London-based homeless charity and advocacy organisation Groundswell. The details of the development of this intervention are presented in Additional file 2.

### Outcomes and follow-up

The primary outcome of interest was successful achievement of an appropriate clinical endpoint, defined as engagement with clinical hepatitis services i.e. three engagements within 6 months of the first booked clinical appointment. An engagement could be a review with a doctor or nurse, FibroScan or ultrasound scan, or a blood test. This number was set after consultation with clinical colleagues to demonstrate a real willingness of the participant to engage.

The secondary trial outcomes for enrolled participants were (a) successfully reaching a sustained virological response (SVR; an undetectable viral load for 6 months after the end of treatment) against HCV and (b) successful engagement, reaching a full clinical diagnosis, or commencing treatment for HBV.

Outcome data collection and the process for withdrawals are described in more detail in Additional file 2.

### Statistical analysis

Data collection and cleaning are described in detail in Additional file 2.

Following descriptive analyses of the baseline population, absolute differences in the proportion of participants successfully achieving three engagements in the intervention and control arms were calculated as an intention-to-treat analysis using a generalised linear model assuming a binomial distribution and an identity link function. The results of this model were compared to that of a logistic regression model. Additional details about the building of these models are presented in Additional file 2.

After examining the distribution of individuals who withdrew or became lost to follow-up (LFU), a post hoc per protocol sensitivity analysis was planned where people in the intervention arm who withdrew or became LFU before they had a peer advocate assigned were re-categorised into the standard of care arm.

Descriptive analyses were undertaken for the secondary outcomes of interest, due to the small numbers of relevant patients.

## Results

### Baseline study population

**Fig. 1:**
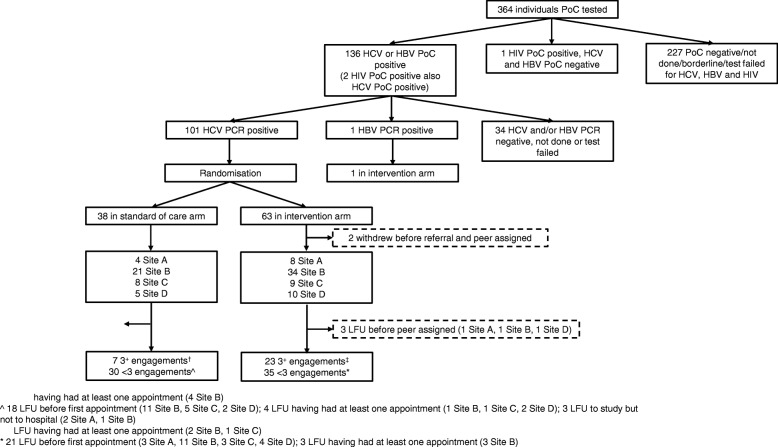
Flow chart of participation

Between 15 August 2013 and 10 June 2015, 364 individuals across a total of 27 outreach services in London consented to point-of-care testing (Fig. 1). Recruitment was stopped when we reached our sample size, allowing for delays in obtaining confirmatory PCR results. Follow-up was completed on 29 April 2016. The baseline characteristics of the individuals who consented are presented in Table 1.

**Table 1:** Baseline characteristics of consenting participants

Characteristic	Overall		Enrolled^		Intervention^		
	No.	Col. %	No.	Col. %	No.	Col. %	*p*
Overall	364	100.0	101	100.0	63	100.0	
Sex							0.45
Male	278	76.4	81	80.2	52	82.5	
Female	86	23.6	20	19.8	11	17.5	
Missing	0	0.0	0	0.0	0	0.0	
Age categorised (years)							0.58
16–25	22	6.0	1	1.0	1	1.6	
26–35	79	21.7	16	15.8	10	15.9	
36–45	130	35.7	42	41.6	23	36.5	
46–55	105	28.8	35	34.7	25	39.7	
56–65	27	7.4	6	5.9	3	4.8	
66–75	1	0.3	1	1.0	1	1.6	
Missing	0	0.0	0	0.0	0	0.0	
Ethnicity							0.59
White other	173	47.5	70	69.3	42	66.7	
White central/eastern European	85	23.4	9	8.9	6	9.5	
Indian subcontinent	5	1.4	1	1.0	0	0.0	
Black	72	19.8	12	11.9	9	14.3	
Mixed/other	27	7.4	8	7.9	6	9.5	
Missing	2	0.5	1	1.0	0	0.0	
UK born							> 0.99
No	88	24.2	23	22.8	14	22.2	
Yes	276	75.8	78	77.2	49	77.8	
Missing	0	0.0	0	0.0	0	0.0	
Use of illicit drugs							0.56
Absent	69	19.0	1	1.0	1	1.6	
Present, but unknown what/when	1	0.3	21	20.8	14	22.2	
Present, previous	81	22.3	0	0.0	0	0.0	
Present, current non-injecting	131	36.0	47	46.5	26	41.3	
Present, current injecting	82	22.5	32	31.7	22	34.9	
Missing	0	0.0	0	0.0	0	0.0	
Homelessness							0.92
Absent	51	14.0	15	14.9	10	15.9	
Present, previous	112	30.8	51	50.5	32	50.8	
Present, current	200	54.9	35	34.7	21	33.3	
Missing	1	0.3	0	0.0	0	0.0	
Imprisonment							0.34
Absent	128	35.2	16	15.8	10	15.9	
Present, > 5 years ago	103	28.3	47	46.5	33	52.4	
Present, ≤ 5 years ago	132	36.3	37	36.6	20	31.7	
Missing	1	0.3	1	1.0	0	0.0	
Alcohol-related concerns							0.68
Absent, not sure or missing	148	40.7	45	44.6	27	42.9	
Present	216	59.3	56	55.4	36	57.1	
Smoking							–*
Current	321	88.2	100	99.0	62	98.4	
Ex-smoker	14	3.8	0	0.0	0	0.0	
Missing	29	8.0	1	1.0	1	1.6	
HIV status							> 0.99
Negative or not sure	360	98.9	99	98.0	62	98.4	
Positive	4	1.1	2	2.0	1	1.6	
HBV vaccination status							0.81
Not vaccinated or not sure	137	37.6	24	23.8	16	25.4	
One or more doses	227	62.4	77	76.2	47	74.6	
Previous testing							0.29
Not tested or not sure	92	25.3	4	4.0	4	6.3	
Yes, for HBV or HCV	272	74.7	97	96.0	59	93.7	
Previous diagnosis							0.79
Not diagnosed or not sure	228	62.6	19	18.8	11	17.5	
Yes, for HBV, HCV or liver disease	136	37.4	82	81.2	52	82.5	
‘Known positive’ at the time of recruitment							0.54
No	310	85.2	47	46.5	31	49.2	
Yes	54	14.8	54	53.5	32	50.8	
HCV point-of-care test result							
Negative	224	61.5					
Positive	136	37.4					
Not done (refused)	3	0.8					
Borderline	1	0.3					
HBV point-of-care test result							
Negative	306	84.1					
Positive	3	0.8					
Not done (refused)	46	12.6					
Test failed	9	2.5					
HIV point-of-care test result							
Negative	334	91.8					
Positive	3	0.8					
Not done (refused)	27	7.4					

^Excluding one individual positive for HBV but not HCV at confirmatory testing. *Everyone in both randomisation arms with information available was a current smoker. *Col* column, *HBV* hepatitis B virus, *HCV* hepatitis C virus, *p* Fisher’s exact test *p* value (missing values excluded) comparing individuals in the intervention and standard of care arms

Consenting participants were largely male (278/364, 76.4%), with a median age of 43 years (interquartile range 35–48), born in the UK (276/364, 75.8%) and of White other or White central/eastern European ethnicity (258/364, 76.4%). High levels of key social risk factors associated with being at risk for HCV infection were displayed, including 82/364 (22.5%) of participants being current injecting users of illicit drugs. The majority of participants were active smokers (321/364, 88.2%). Around three quarters (272/364) had previously been tested for HCV or HBV and over a third previously diagnosed with HBV, HCV or another form of liver disease (136/364). Fifty-four individuals (14.8%) were recruited as ‘known positives’ from services.

Of these 364 individuals, 136 had a positive point-of-care test for HCV, three for HBV and three for HIV. Chronic HBV infection was confirmed in one individual by PCR testing, who was enrolled and placed in the intervention arm. One hundred one individuals were enrolled with a chronic HCV infection by PCR. This population was similar to the overall study population, but a greater proportion of individuals were from a White other ethnic background (70/101, 69.3%), current users of illicit drugs (79/101, 78.2% versus 213/364, 58.5%) or displayers of other social risk factors; had been previously tested or diagnosed; or were ‘known positives’ (54/101, 53.5% versus 54/364, 14.8%).

Sixty-three individuals were randomised to receive the intervention and 38 to the standard of care arm (Fig. 1). The distribution of baseline characteristics was not found to be appreciably different between randomisation arms among the 101 individuals positive for a chronic HCV infection who were enrolled (Table 1).

### Withdrawals and losses to follow-up

Three individuals withdrew post-randomisation during the study, one from the standard of care and two from the intervention arm (Fig. 1). The characteristics of those who withdrew were not substantially different from those who did not (Additional file 3). Fifty-nine individuals were LFU, three of whom were in the intervention arm and whom became LFU before a peer advocate was assigned (Fig. 1). Their characteristics were not substantially different from the overall enrolled population, apart from when it came to a history of imprisonment: more had been imprisoned in the last 5 years (Additional file 3).

### Successful engagement with clinical services

50.5% of the HCV-positive individuals (51/101) engaged with services at least once within 6 months of their first booked clinical appointment (median one, maximum six, interquartile range 0–3). 29.7% (30/101) achieved a successful outcome, i.e. engaged with services three times or more. Of these 30 individuals, seven were in the standard of care arm (7/38, 18.4%) and 23 (23/63, 36.5%) were in the intervention arm. Their baseline characteristics are documented in Table 2.

**Table 2:** Outcomes by study arm and baseline characteristics

Characteristic	≥ 3 engagements	
	No.	Row %
Overall	30	29.7
Randomisation		
Standard of care	7	18.4
Intervention	23	36.5
Sex		
Male	28	34.6
Female	2	10.0
Missing	0	–
Age categorised (years)		
16–25	0	0.0
26–35	3	18.8
36–45	12	28.6
46–55	15	42.9
56–65	0	0.0
66–75	0	0.0
Missing	0	–
Ethnicity		
White other	18	25.7
White central/eastern European	1	11.1
Indian subcontinent	0	0.0
Black	7	58.3
Mixed/other	3	37.5
Missing	0	0.0
UK born		
No	5	21.7
Yes	25	32.1
Missing	0	–
Use of illicit drugs		
Absent	0	0.0
Present, but unknown what/when	3	14.3
Present, previous	0	–
Present, current non-injecting	16	34.0
Present, current injecting	11	34.4
Missing	0	–
Homelessness		
Absent	7	46.7
Present, previous	15	29.4
Present, current	0	0.0
Missing	8	–
Imprisonment		
Absent	3	18.8
Present, > 5 years ago	18	38.3
Present, ≤ 5 years ago	9	24.3
Missing	0	0.0
Alcohol-related concerns		
Absent, not sure or missing	14	31.1
Present	16	28.6
Smoking		
Current	30	30.0
Ex-smoker	0	–
Missing	0	0.0
HIV status		
Negative or not sure	30	30.3
Positive	0	0.0
HBV vaccination status		
Not vaccinated or not sure	8	33.3
One or more doses	22	28.6
Previous testing		
Not tested or not sure	1	25.0
Yes, for HBV or HCV	29	29.9
Previous diagnosis		
Not diagnosed or not sure	5	26.3
Yes, for HBV, HCV or liver disease	25	30.5
‘Known positive’ at the time of recruitment		
No	11	23.4
Yes	19	35.2

Excluding one individual positive for HBV but not HCV at confirmatory testing. *HBV* hepatitis B virus, *HCV* hepatitis C virus

Outcomes by peer advocate were investigated as part of the process to determine the potential need to adjust for clustering during the regression analysis. Of the 63 individuals in the intervention arm, five were not assigned a peer advocate, two due to withdrawing and three due to becoming LFU before assignment could occur. Of the remaining 58 individuals, peer advocate A was assigned to the greatest number (33/58, 56.9%; Additional file 4). Although there was variation in terms of the proportions of individuals cared for by each peer advocate that reached a successful outcome, the confidence intervals (CIs) around each percentage overlapped with the overall percentage.

Univariable regression models were thus built without adjustment for clustering by peer advocate to examine the relationship between the intervention and successfully engaging. In the model of absolute differences, patients in the intervention arm were found to have an 18.1% (95% CI 1.0%–35.2%, *p* value = 0.04) increased likelihood of a successful treatment outcome versus those in the standard of care arm. In the model of relative differences, the odds of reaching a successful treatment outcome were 2.55 times higher (95% CI 0.97–6.70, *p* = 0.06) among individuals in the intervention arm versus those in the standard of care arm.

Although imbalances by randomisation arm had not been detected in baseline characteristics, bivariable models adjusting for potential confounders were created. Point estimates differed (Additional file 5), but the CIs for each model overlapped with that of the univariable model for both absolute and relative measures. A Lowess plot of age versus outcomes revealed a non-linear relationship and thus both age and age squared were included in the relevant model. Imprisonment and sex had particularly large impacts in terms of drawing the CIs towards the null. Smoking and HIV status were collinear with the outcome and thus could not be assessed.

There was no evidence of effect modification by any of the predefined variables. Tests for interaction failed for ethnicity, being UK born, use of illicit drugs and previous testing due to small numbers in particular strata.

### Sensitivity analyses

Ad hoc per protocol analyses were undertaken for both the absolute and relative models to determine the impact of initial non-assignation of a peer advocate to individuals in the intervention arm due to withdrawal or LFU. Assigning affected individuals to the standard of care arm, the absolute difference in the percentage likelihood of achieving a successful treatment outcome increased to 23.4% (95% CI 6.6–40.1%, *p* = 0.01) and the relative difference to 3.38 (95% CI 1.29–8.88, *p* = 0.01).

### Secondary outcomes

Among individuals with a chronic HCV infection, none achieved a SVR during the study period. The individual diagnosed with chronic HBV assigned to the intervention arm did not achieve a successful outcome of three or more engagements with healthcare services within six months; they became LFU eight months after their first appointment (with no subsequent appointments).

### Adverse events

No serious adverse events were documented within the study.

## Discussion

In a RCT of an individual-level, community-controlled, peer support intervention to promote successful engagement with clinical services for individuals diagnosed with chronic HCV, we demonstrate that providing patients with a peer advocate increased their absolute likelihood of successfully engaging with healthcare systems by 18.1%. In a logistic regression model, this translated into 2.55 times the odds of a successful outcome, albeit with a CI that minimally crossed the null.

Importantly, an ad hoc per protocol sensitivity analysis revealed the impact of not staying within the study for long enough to be assigned a peer advocate. Within this population, arrest and imprisonment were key reasons for becoming LFU. When individuals in the intervention arm who were never assigned a peer advocate were analysed as though they were in the standard of care arm both effect sizes and statistical measures of association were strengthened.

We believe this to be the first RCT to evaluate the efficacy of a peer support intervention at improving engagement with healthcare services for chronic HCV. The closest previous study found in the literature was a randomised pilot trial in Belgium of an educational intervention that included a peer component and use of FibroScan screening [27]. This study was small (52 participants) and did not detect an enduring change in willingness to be treated in the intervention versus control arms. A number of descriptive observational studies have documented the role of different peer models in HCV testing and treatment [17, 19, 22–25], but provided no formal proof of the causal effects of the implemented interventions.

Some limitations of the study should be considered. The study was not blinded, meaning that participants were aware of the trial arm that they were assigned to, as were study staff. This could have altered participant behaviour and the way that the outcome was documented during data collection, biasing the effect estimate away from the null, although data collection was undertaken using standardised forms and processes across all patients. Data were only collected from the hospitals that the patients initially chose to attend. It is possible that patients in the standard of care arm in particular changed hospital during their engagement with services, biasing the effect estimate away from the null. Given that such patients would have likely had to re-start their diagnostic process from scratch, however, this can still be seen as a failure within the clinical system. Originally, only three peer advocates were planned to be involved in the intervention arm, but eight were actually utilised. As we found no evidence of clustering by peer, this is unlikely to have reduced study power. Our peer advocates were all male, but we found no evidence for effect modification by participant sex within our analysis. No patients achieved a SVR during the study, partly due to delays placing individuals on treatment as a result of changes in the regimens that were available within national healthcare services during the RCT. During the trial, treatment was additionally limited to those with higher FibroScan scores. This prevented us from analysing the impact of peer support on treatment success rates.

Since 2016, ODNs have been responsible for delivering HCV treatment in England, with monthly treatment targets that should promote access to DAAs for marginalised individuals. This expansion of treatment comes with its own challenges regarding ensuring patient engagement with the diagnostic and treatment pathway. This study provides high-quality evidence that peer advocates may provide an efficacious way of promoting this, in line with global calls for the use of peer support models in chronic HCV [26].

## Conclusions

In conclusion, our findings indicate that peer support can improve the engagement of patients with chronic HCV with healthcare services. The cost-effectiveness of such interventions in the relevant populations should be assessed before they are implemented in different healthcare settings.

## Additional files

Additional file 1: Study questionnaire. Copy of questionnaire used during study. (DOCX 771 kb)

Additional file 2: Detailed methodology. Detailed methods for the trial. (DOCX 27 kb)

Additional file 3: Baseline characteristics of individuals who withdrew or were lost to follow up. Tabulated baseline information for study participants who were lost to follow-up or who withdrew. (DOCX 22 kb)

Additional file 4: Outcomes by peer advocate. Tabulated study outcomes, stratified by peer advocate. (DOCX 18 kb)

Additional file 5: Regression analysis of effectiveness of peer support intervention in Hepatitis C Virus-positive individuals, adjusted for potential confounding. Tabulated additional analyses. (DOCX 19 kb)

## Abbreviations

CI: Confidence interval
DAAs: Directly acting antivirals
GP: General practitioner (primary care provider)
HBV: Hepatitis B virus
HCV: Hepatitis C virus
HHPA: Homeless Health Peer Advocacy
LFU: Lost to follow-up
ODN: Operational Delivery Network
PCR: Polymerase chain reaction
PHE: Public Health England
RCT: Randomised controlled trial
SVR: Sustained virological response
