# Supplementary figures and images for: Improving engagement with healthcare in hepatitis C: a randomised controlled trial of a peer support intervention
Source: BMC Med. 2019 Apr 1;17:71. doi: 10.1186/s12916-019-1300-2 (PMC6442435; doi:10.1186/s12916-019-1300-2)

**ADDITIONAL FILE 1: Study questionnaire**

**
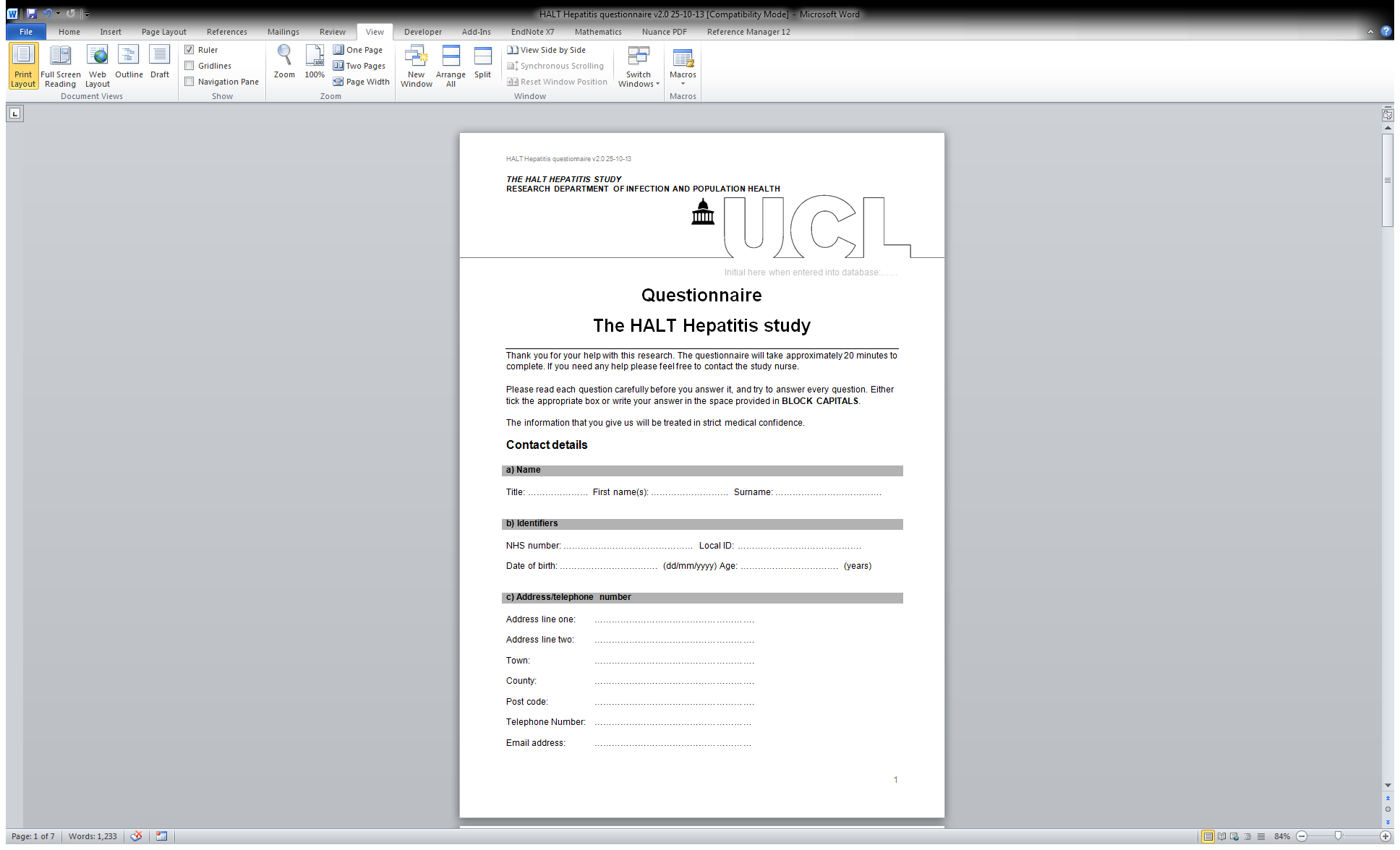
**

**
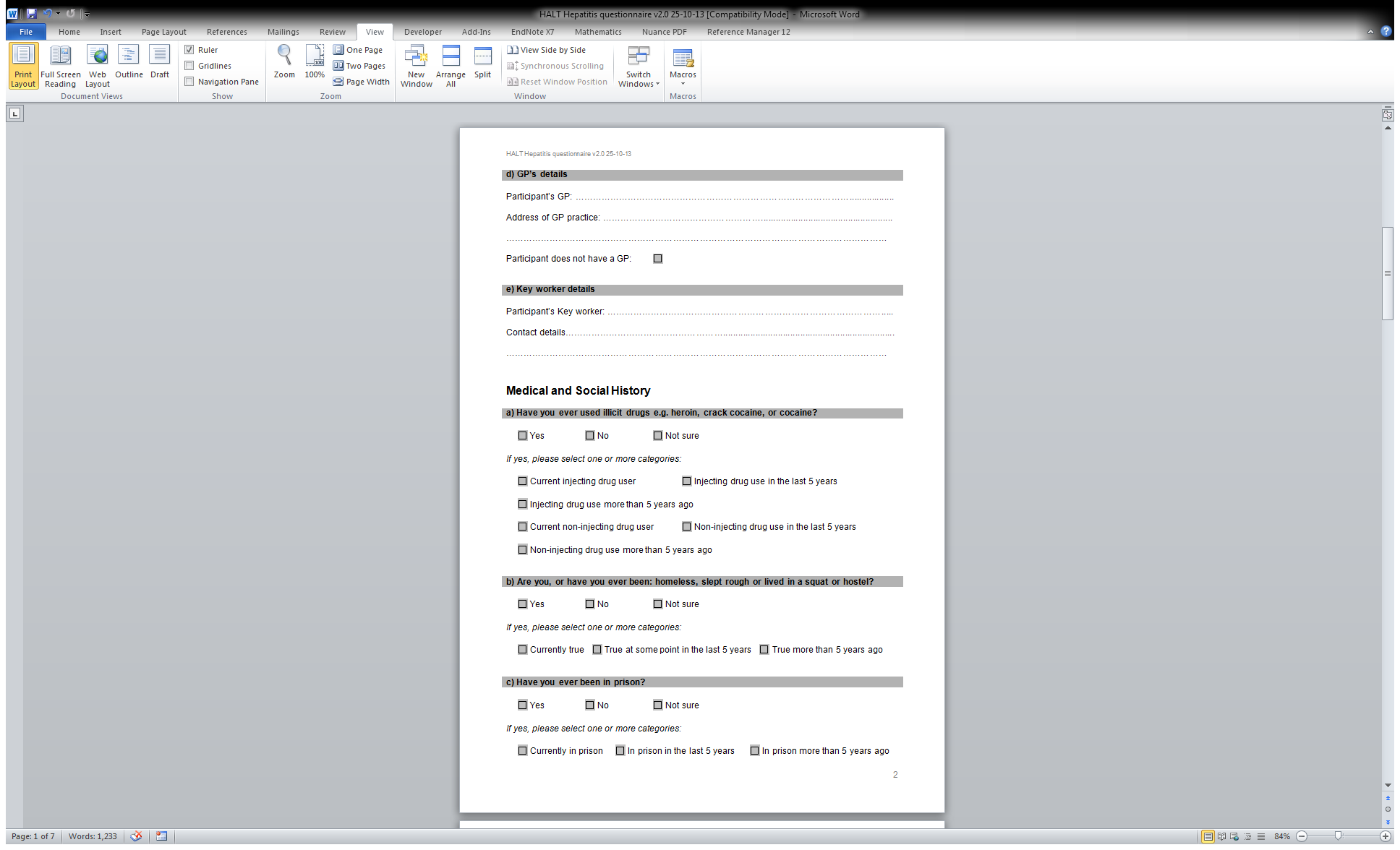
**

**
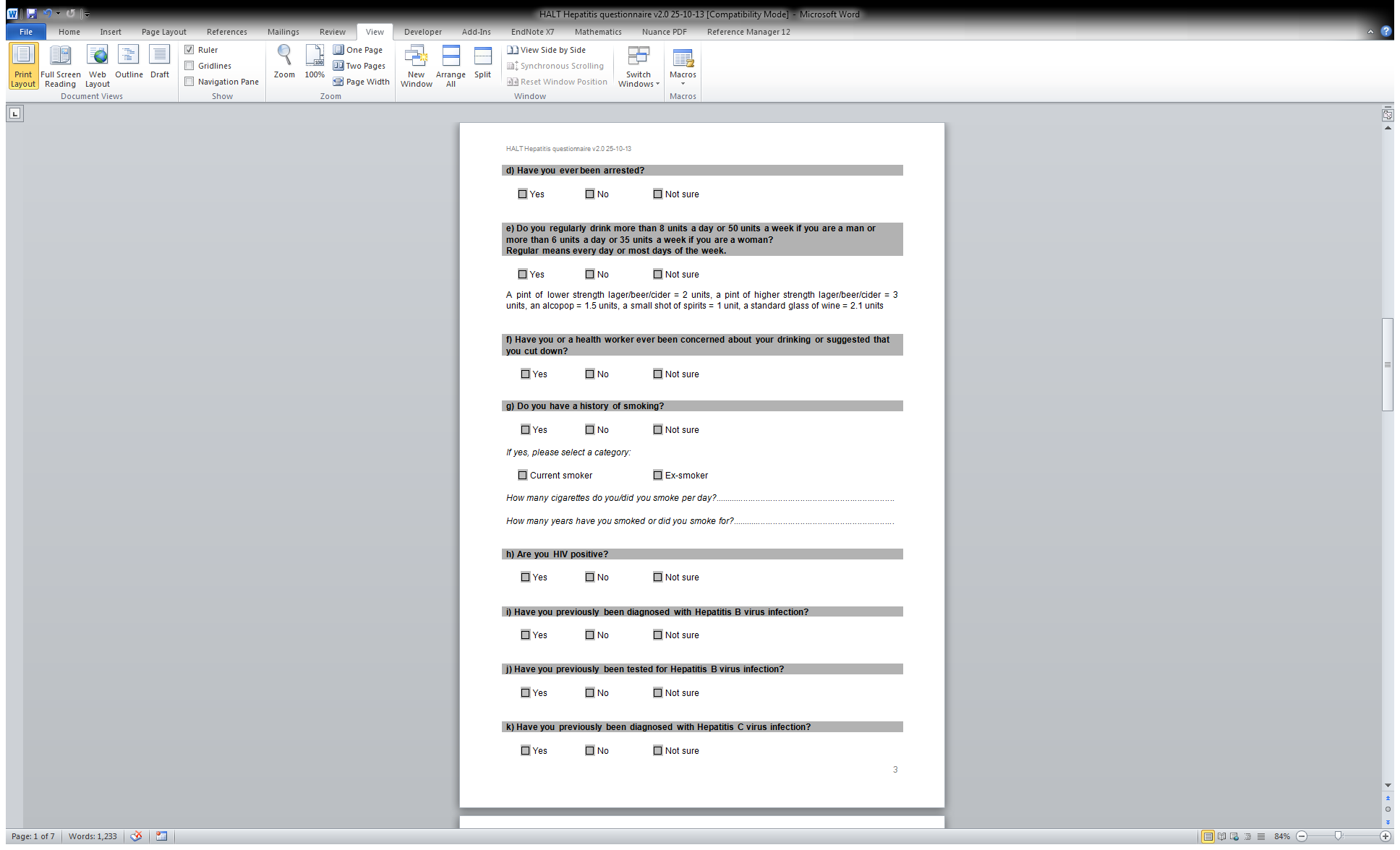
**

**
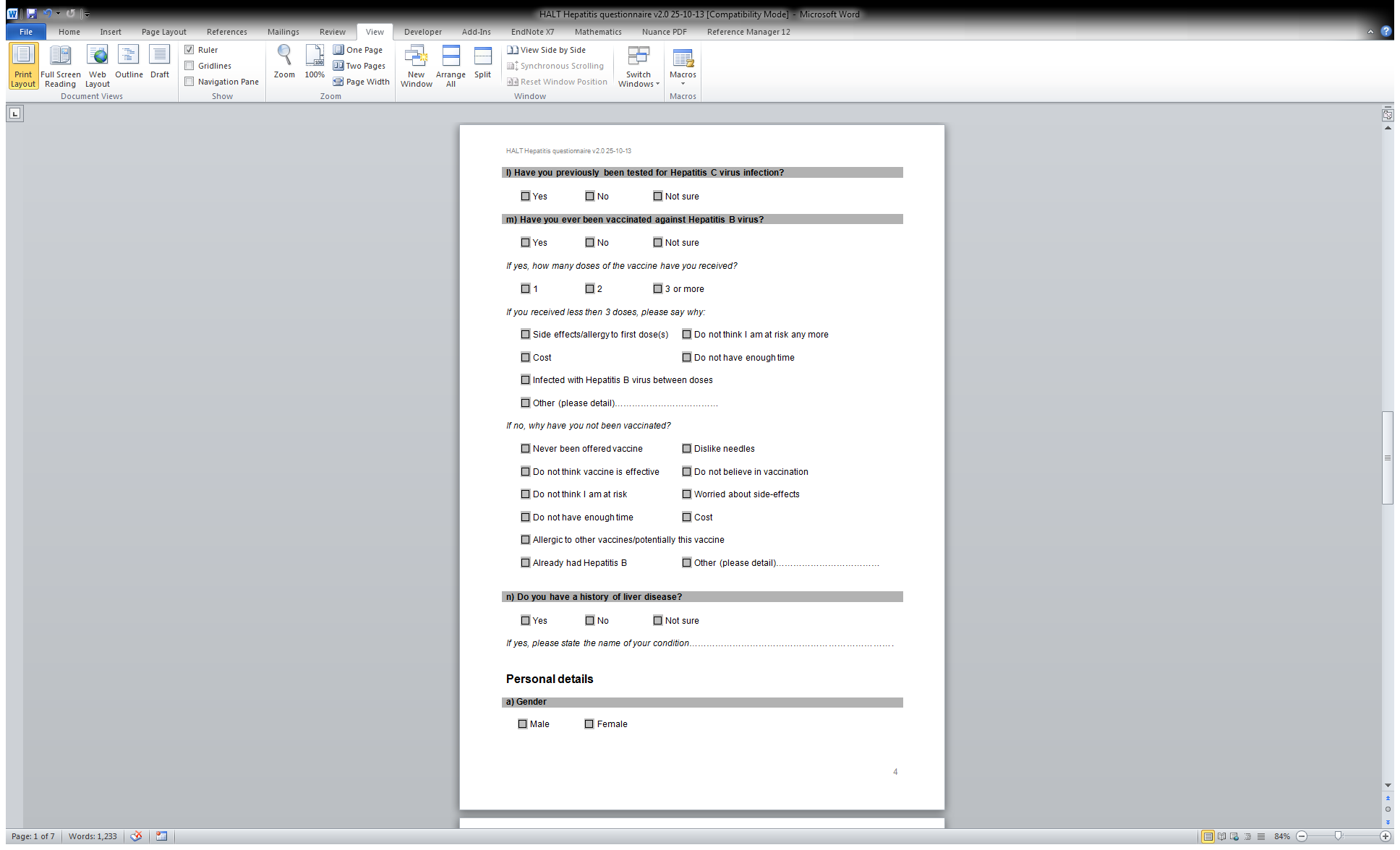
**

**
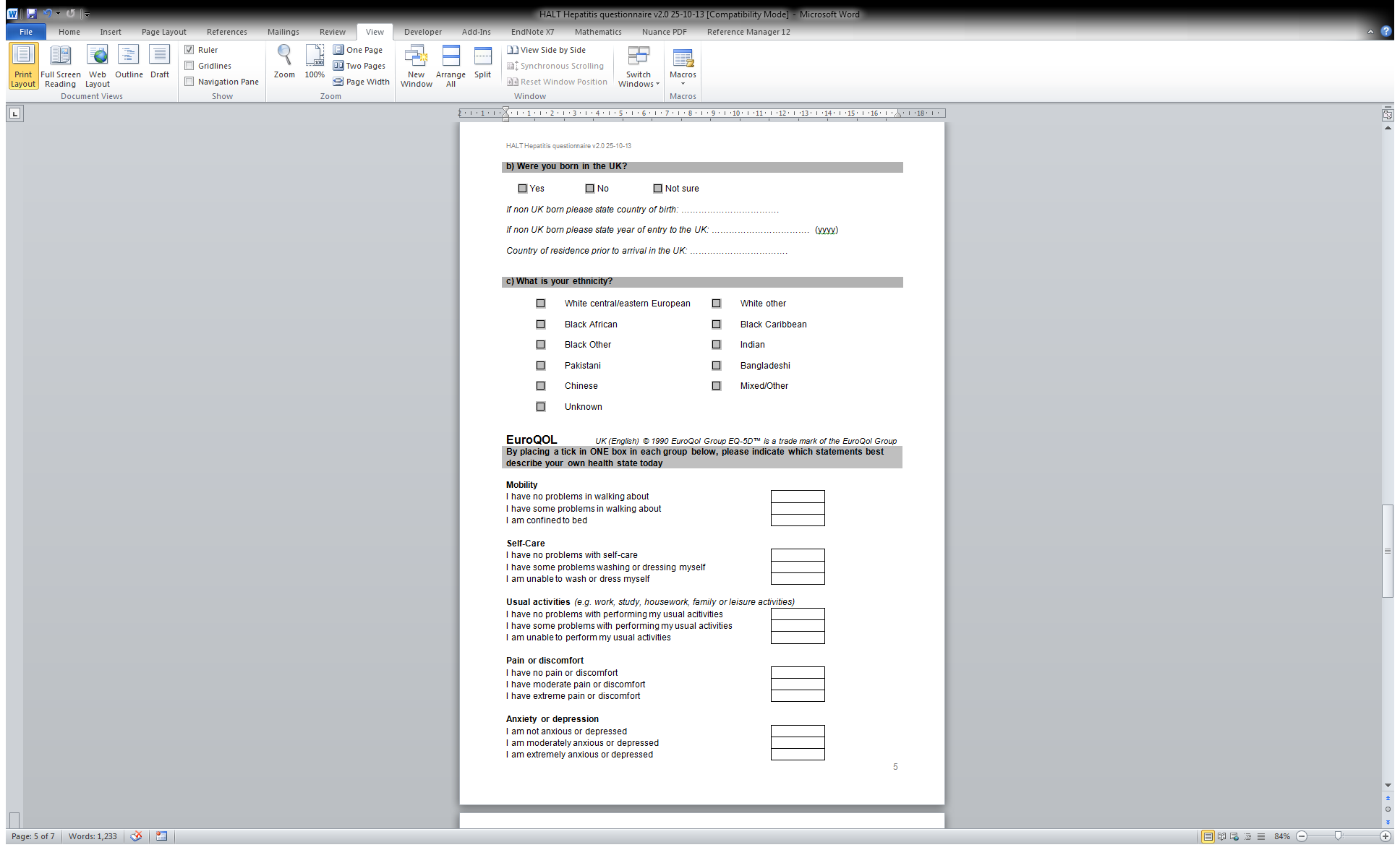
**

**
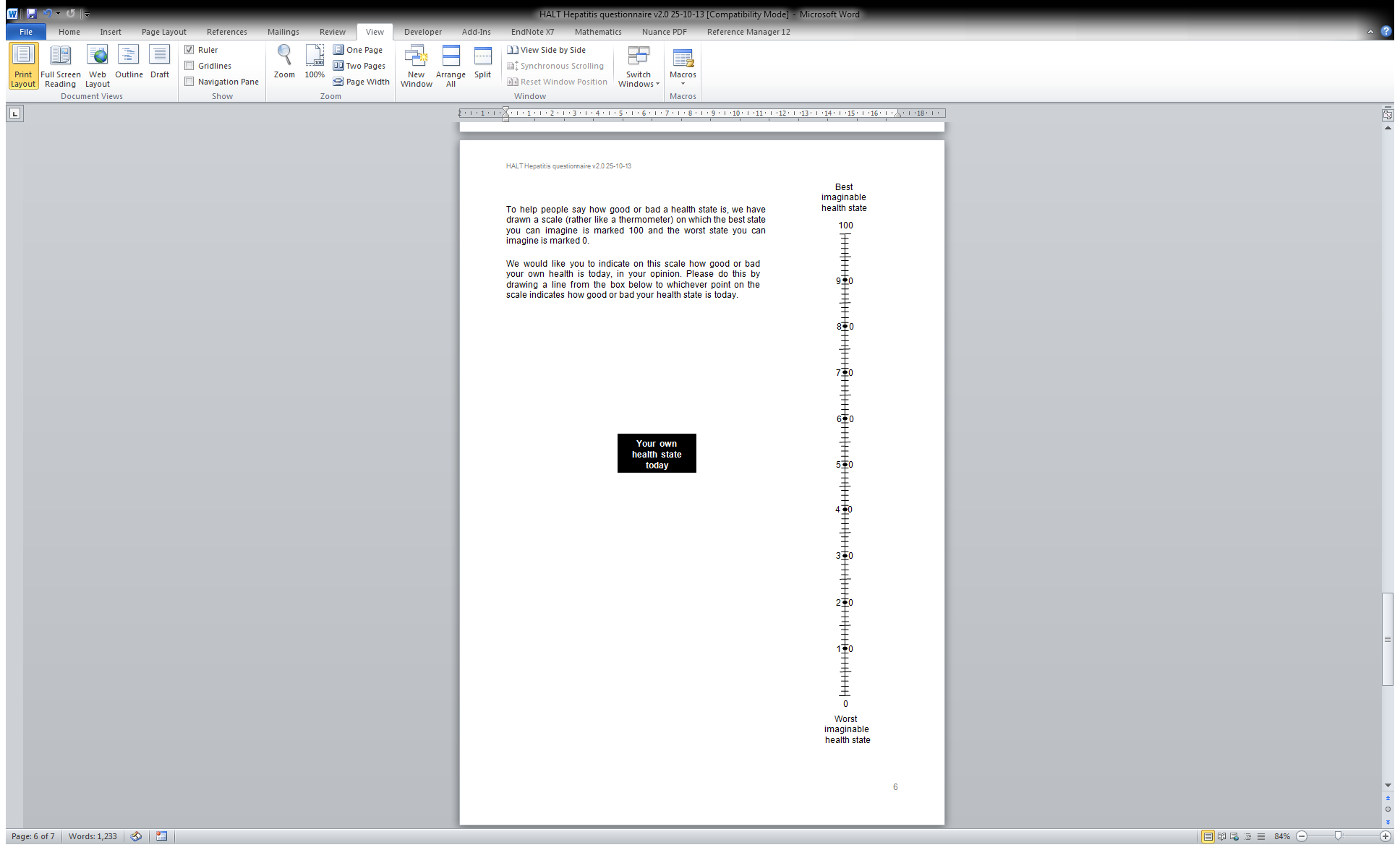
**

**
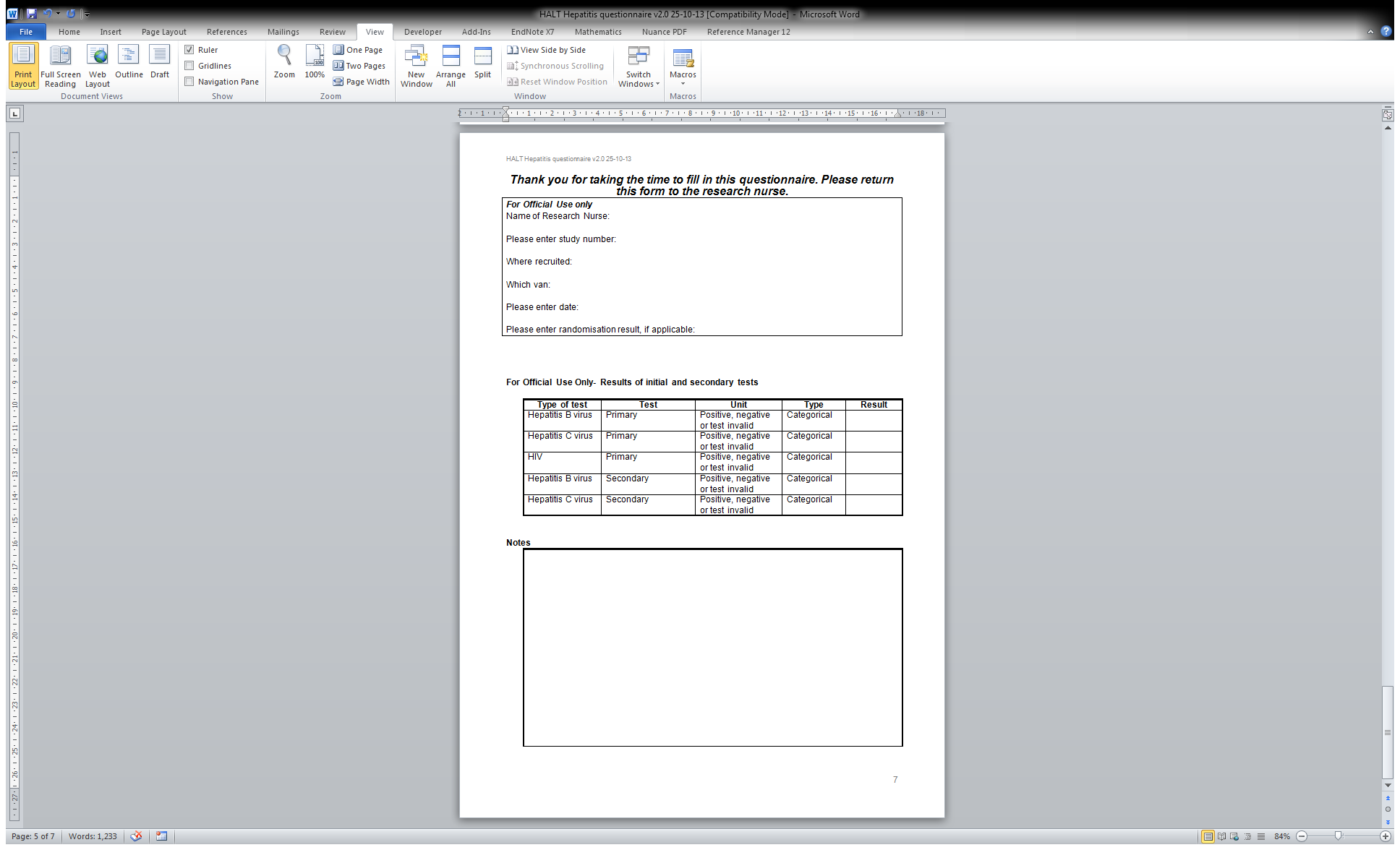
**

Supplement: Supplementary file 1 — Study questionnaire. Copy of questionnaire used during study. (DOCX 771 kb) [file 12916_2019_1300_MOESM1_ESM.docx]
