# Supplementary material for: Improving engagement with healthcare in hepatitis C: a randomised controlled trial of a peer support intervention
Source: BMC Med. 2019 Apr 1;17:71. doi: 10.1186/s12916-019-1300-2 (PMC6442435; doi:10.1186/s12916-019-1300-2)
Supplement: Supplementary file 2 — Detailed methodology. Detailed methods for the trial. (DOCX 27 kb) [file 12916_2019_1300_MOESM2_ESM.docx]

# ADDITIONAL FILE 2: Detailed methods

## Randomisation

The individual-level randomisation sequence was generated by Sealed Envelope using their standard computer algorithms without blocking, such that the sequence was concealed to the study team until the interventions were assigned. Individuals testing positive for hepatitis B virus (HBV) (including those positive for both hepatitis C virus [HCV] and HBV) were automatically assigned to the intervention arm. The nature of the Peer Support intervention meant that blinding was not possible for participants.

**Intervention**

Groundswell has provided an award-winning, previously evaluated, Homeless Health Peer Advocacy (HHPA) service since 2010.[1] HHPA provides one-to-one support for homeless people to attend health appointments; overcoming the practical, personal and systemic barriers preventing access to healthcare. Delivered by formerly homeless volunteers who can build trusting relationships with homeless people who others find ‘hard-to-reach’, HHPA aims to increase adherence to treatment and reduce missed appointments, unplanned admissions to hospital or the use of Accident and Emergency units.

As part of a process of co-design, focus groups of chronic HCV patients and professionals from drug and alcohol, homelessness, and health services tailored the HHPA service to the needs of the relevant population group for the trial. A model was developed where, in addition to accompanying people to appointments, Peer Advocates did a range of work to promote engagement. Contact was made with clients between appointments by telephone, texts and regularly meetings. Peer Advocates directly contacted hospitals for news of appointment dates, acted as a permanent address to receive appointment letters, and supported people to tackle their other health issues. Advocates provided practical assistance only around health issues, but they were also able to provide signposting to other agencies for support with benefits, housing and legal issues that prevented clients from proactively engaging with their healthcare. The Peer Advocates’ perspective on the work that they undertook has been previously qualitatively analysed and revealed the importance of self-disclosure within the Peer Advocate-client relationship.[2]

In line with the HHPA model, Peer Advocates were people with lived knowledge similar to the individuals recruited into the intervention and control arms. They had experienced homelessness and many had a history of substance misuse, mental health needs and imprisonment. Peer Advocates also had a similar age, ethnicity and country of birth (UK versus non-UK born) distribution as our recruited population. Achieving the same sex distribution was more challenging, as the demographics of the Peer Advocates reflected those of the population that we recruited from, which was highly male. All Peer Advocates were thus male, although within Groundswell they had female colleagues available for consultation. Peers Advocates were a mixture of full and part-time. Full-time individuals were paid; part-time individuals undertook the role on a voluntary basis whilst complying with UK regulations on the entitlement to benefits. Out-of-pocket expenses incurred directly as a result of the study were remunerated e.g. travel to meet with a participant in the intervention arm.

Peer Advocates attended a comprehensive six week training programme, including modules on boundary setting, safe guarding, information governance, working with vulnerable adults and blood-borne viruses (the latter delivered by the Hepatitis C Trust). New Peer Advocates initially shadowed an experienced Peer. They had a designated line manager throughout the study, monthly group supervision sessions, ‘one on ones’, and ongoing training, in addition to clinical support by the study team, as appropriate.

**Outcomes and follow-up**

At the end of the study the clinical notes for each patient were accessed at the hospital they were referred to and outcome information recorded into the study database by the Research Nurses, who were not blinded to the randomisation arm. To allow every enrolled participant to achieve at least six months of follow-up after their first booked appointment, engagements were recorded up until the 29^th^ April 2016.

Participants were able to withdraw from the study at any time by notifying the study team. Their data was included within the study up until the date of withdrawal. As well as explicitly withdrawing, patients could also become lost to follow up (LFU; i.e. uncontactable by the study team) at any stage. If this was after the patient was referred, this did not necessarily mean that they lost contact with clinical services.

**Sample size**

To detect a clinically important 30% increase in individuals engaging with healthcare services from a baseline of 10%, with 80% power and an alpha of 5%, we planned to enrol 38 HCV positive individuals per arm. This number was inflated to 61 in the intervention arm, to account for anticipated clustering by Peer Advocate. Pilot data from the early stages of another study within London initially indicated a 15% prevalence of HCV infection.[3] In order to enrol 99 HCV positive individuals, we thus planned to screen 660 consenting participants.

**Statistical analysis**

Data for enrolled participants were entered into a standardised, pre-defined, Microsoft Access database. A separate database was used to record data for the non-enrolled individuals. A randomly selected 10% of records across both databases were double entered and checked for consistency. Sense checks were also performed within each record. Data were exported into Stata 15 for analysis, where they were cleaned and categorised. All of the following variables were self-reported. A participant’s use of illicit drugs was classified as absent and present, the latter either as unknown what was used and when, previous usage, current non-injecting drug use, and current injecting drug use. A participant’s homelessness status was categorised as absent or present, the latter either as previous or current. A participant’s imprisonment status was categorised as absent or present, the latter either as more than 5 years ago or less than or equal to five years ago. Alcohol-related concerns was a composite variable derived from two questions about levels of drinking. The first asked about regular drinking exceeding eight units a day/50 units a week (males) or six units a day/35 units a week (females). The second asked if the individual in question or a health worker had ever been concerned about their drinking or suggested reductions in the amount consumed. An answer in the affirmative for either question was classified as present; due to the combining of two questions the remainder of patients had their status classified as absent, not sure, or missing. Participant smoking status was grouped as current or ex-smoker.

HCV, HBV and HIV point-of-care test results were recorded as negative, positive, not done (refused), borderline, or test failed.

During model building, the need to adjust for clustering by Peer Advocate was ascertained by examining the distribution of outcomes by Peer Advocate, and by fitting a model with a sandwich estimator of the variance covariance matrix and comparing that with a model without adjustment for clustering. For the latter, people without a Peer Advocate were treated individually rather than categorised as a ‘no Peer Advocate’ group. Any imbalances in baseline characteristics were ascertained in order to determine the need to adjust for potential confounders. Age, sex, ethnicity, being UK born, use of illicit drugs, homelessness, imprisonment, alcohol use, having previously been tested or diagnosed, or being a ‘known positive’ for HCV were investigated as potential effect modifiers.

**References**

1. Finlayson S, Boelman V, Young R, Kwan A. Saving Lives, Saving Money. How Homeless Health Peer Advocacy Reduces Health Inequalities. 2016.

2. MacLellan J, Surey J, Abubakar I, Stagg HR, Mannell J. Using peer advocates to improve access to services among hard-to-reach populations with hepatitis C: a qualitative study of client and provider relationships. Harm Reduct J. **2017 Nov 28**;14(1):76.

3. Aldridge RW, Hayward AC, Hemming S, Yates SK, Ferenando G, Possas L, et al. High prevalence of latent tuberculosis and bloodborne virus infection in a homeless population. Thorax. **2018 Jun**;73(6):557-64.
