# Supplementary material for: Improving engagement with healthcare in hepatitis C: a randomised controlled trial of a peer support intervention
Source: BMC Med. 2019 Apr 1;17:71. doi: 10.1186/s12916-019-1300-2 (PMC6442435; doi:10.1186/s12916-019-1300-2)
Supplement: Supplementary file 3 — Baseline characteristics of individuals who withdrew or were lost to follow up. Tabulated baseline information for study participants who were lost to follow-up or who withdrew. (DOCX 22 kb) [file 12916_2019_1300_MOESM3_ESM.docx]

**ADDITIONAL FILE 3: Baseline characteristics of individuals who withdrew or were lost to follow up**

| **Characteristic** | | | **Withdrawn** | | | **LFU** | | |
| --- | --- | --- | --- | --- | --- | --- | --- | --- |
|  |  |  | **No.** | **Col. %** | **p-value** | **No.** | **Col. %** | **p-value** |
| **Overall** | |  | 3 | 100.0 |  | 59 | 100.0 |  |
| Sex | | |  |  | >0.99 |  |  | 0.08 |
|  | Male | | 3 | 100.0 |  | 233 | 74.6 |  |
|  | Female | | 0 | 0.0 |  | 71 | 25.4 |  |
|  | Missing | | 0 | 0.0 |  | 0 | 0.0 |  |
| Age categorised (years) | | |  |  | >0.99 |  |  | 0.44 |
|  | 16-25 | | 0 | 0.0 |  | 21 | 1.7 |  |
|  | 26-35 | | 0 | 0.0 |  | 68 | 18.6 |  |
|  | 36-45 | | 2 | 66.7 |  | 104 | 44.1 |  |
|  | 46-55 | | 1 | 33.3 |  | 88 | 27.1 |  |
|  | 56-65 | | 0 | 0.0 |  | 23 | 6.8 |  |
|  | 66-75 | | 0 | 0.0 |  | 0 | 1.7 |  |
|  | Missing | | 0 | 0.0 |  | 0 | 0.0 |  |
| Ethnicity | | |  |  | 0.66 |  |  | 0.73 |
|  | White other | | 2 | 66.7 |  | 132 | 69.5 |  |
|  | White central/eastern European | | 0 | 0.0 |  | 78 | 10.2 |  |
|  | Indian subcontinent | | 0 | 0.0 |  | 4 | 1.7 |  |
|  | Black | | 1 | 33.3 |  | 67 | 8.5 |  |
|  | Mixed/other | | 0 | 0.0 |  | 22 | 8.5 |  |
|  | Missing | | 0 | 0.0 |  | 1 | 1.7 |  |
| UK born | | |  |  | 0.13 |  |  | 0.82 |
|  | No | | 2 | 66.7 |  | 74 | 76.3 |  |
|  | Yes | | 1 | 33.3 |  | 230 | 23.7 |  |
|  | Missing | | 0 | 0.0 |  | 0 | 0.0 |  |
| Use of illicit drugs | | |  |  | 0.79 |  |  | 0.35 |
|  | Absent | | 0 | 0.0 |  | 68 | 1.7 |  |
|  | Present, but unknown what/when | | 0 | 0.0 |  | 1 | 0.0 |  |
|  | Present, previous | | 1 | 33.3 |  | 71 | 15.3 |  |
|  | Present, current non-injecting | | 1 | 33.3 |  | 102 | 49.2 |  |
|  | Present, current injecting | | 1 | 33.3 |  | 62 | 33.9 |  |
|  | Missing | | 0 | 0.0 |  | 0 | 0.0 |  |
| Homelessness | | |  |  | >0.99 |  |  | 0.89 |
|  | Absent | | 0 | 0.0 |  | 43 | 13.6 |  |
|  | Present, previous | | 2 | 66.7 |  | 81 | 52.5 |  |
|  | Present, current | | 1 | 33.3 |  | 179 | 33.9 |  |
|  | Missing | | 0 | 0.0 |  | 1 | 0.0 |  |
| Imprisonment | | |  |  | 0.56 |  |  | 0.04 |
|  | Absent | | 1 | 33.3 |  | 116 | 20.3 |  |
|  | Present, >5 years ago | | 1 | 33.3 |  | 82 | 35.6 |  |
|  | Present, <=5 years ago | | 1 | 33.3 |  | 106 | 42.4 |  |
|  | Missing | | 0 | 0.0 |  | 0 | 1.7 |  |
| Alcohol-related concerns | | |  |  | 0.58 |  |  | 0.22 |
|  | Absent, not sure, or missing | | 2 | 66.7 |  | 125 | 39.0 |  |
|  | Present | | 1 | 33.3 |  | 179 | 61.0 |  |
| Smoking | | |  |  | -* |  |  | -* |
|  | Current | | 3 | 100.0 |  | 262 | 98.3 |  |
|  | Ex-smoker | | 0 | 0.0 |  | 14 | 0.0 |  |
|  | Missing | | 0 | 0.0 |  | 28 | 1.7 |  |
| HIV status | | |  |  | >0.99 |  |  | >0.99 |
|  | Negative or not sure | | 3 | 100.0 |  | 301 | 98.3 |  |
|  | Positive | | 0 | 0.0 |  | 3 | 1.7 |  |
| HBV vaccination status | | |  |  | 0.14 |  |  | 0.35 |
|  | Not vaccinated or not sure | | 2 | 66.7 |  | 12 | 20.3 |  |
|  | One or more doses | | 1 | 33.3 |  | 47 | 79.7 |  |
| Previous testing | | |  |  | >0.99 |  |  | 0.64 |
|  | Not tested or not sure | | 0 | 0.0 |  | 89 | 5.1 |  |
|  | Yes, for HBV or HCV | | 3 | 100.0 |  | 215 | 94.9 |  |
| Previous diagnosis | | |  |  | >0.99 |  |  | 0.07 |
|  | Not diagnosed or not sure | | 0 | 0.0 |  | 213 | 25.4 |  |
|  | Yes, for HBV, HCV or liver disease | | 3 | 100.0 |  | 91 | 74.6 |  |
| ‘Known positive’ at the time of recruitment | | |  |  | 0.60 |  |  | 0.16 |
|  | No | | 2 | 66.7 |  | 31 | 52.5 |  |
|  | Yes | | 1 | 33.3 |  | 28 | 47.5 |  |

Excluding one individual positive for HBV but not HCV at confirmatory testing. Col- column, HBV- hepatitis B virus, HCV- hepatitis C virus, LFU- lost to follow up, p- Fisher’s exact test p-value (missing values excluded) comparing those who withdrew/were LFU to the rest of the enrolled population.
