# Supplementary material for: Improving engagement with healthcare in hepatitis C: a randomised controlled trial of a peer support intervention
Source: BMC Med. 2019 Apr 1;17:71. doi: 10.1186/s12916-019-1300-2 (PMC6442435; doi:10.1186/s12916-019-1300-2)
Supplement: Supplementary file 4 — Outcomes by peer advocate. Tabulated study outcomes, stratified by peer advocate. (DOCX 18 kb) [file 12916_2019_1300_MOESM4_ESM.docx]

**ADDITIONAL FILE 4: Outcomes by Peer Advocate**

|  | | **Overall** | | **>=3 engagements** | |
| --- | --- | --- | --- | --- | --- |
|  |  | **No.** | **Col. %** | **No.** | **Row % (95% CI)** |
| **Overall** | | 58 | 100.0 | 23 | 39.7 (27.0-53.4) |
| Peer Advocate | |  |  |  |  |
|  | A | 33 | 56.9 | 12 | 36.4 (20.4-54.9) |
|  | B | 8 | 13.8 | 1 | 12.5 (0.3-52.7) |
|  | C | 2 | 3.4 | 1 | 50.0 (1.3-98.7) |
|  | D | 2 | 3.4 | 0 | 0.0 (0.0-84.2)* |
|  | E | 4 | 6.9 | 4 | 100.0 (39.8-100.0)* |
|  | F | 2 | 3.4 | 1 | 50.0 (1.3-98.7) |
|  | G | 7 | 12.1 | 4 | 57.1 (18.4-90.1) |

Excluding one individual positive for HBV but not HCV at confirmatory testing. *- one sided CI, CI- confidence interval
