# Supplementary material for: Improving engagement with healthcare in hepatitis C: a randomised controlled trial of a peer support intervention
Source: BMC Med. 2019 Apr 1;17:71. doi: 10.1186/s12916-019-1300-2 (PMC6442435; doi:10.1186/s12916-019-1300-2)
Supplement: Supplementary file 5 — Regression analysis of effectiveness of peer support intervention in Hepatitis C Virus-positive individuals, adjusted for potential confounding. Tabulated additional analyses. (DOCX 19 kb) [file 12916_2019_1300_MOESM5_ESM.docx]

**ADDITIONAL FILE 5: Regression analysis of effectiveness of Peer SUPPORT intervention in HCV positive individuals, adjusted for potential confounding**

| **Exposure variables** | **Adjusted bivariable regression** | **Adjusted bivariable regression** |
| --- | --- | --- |
|  | Absolute difference (95% CI), p-value | OR (95% CI), p-value |
| Sex | 13.4 (-4.5-31.3), 0.14 | 2.46 (0.92-6.60), 0.07 |
| Age*^†^ | 17.7 (0.3-35.1), 0.05 | 2.51 (0.94-6.70), 0.07 |
| Ethnicity* | 17.9 (1.0-34.8), 0.04 | 2.76 (0.97-7.84), 0.06 |
| UK born | 22.0 (3.6-40.5), 0.02 | 2.55 (0.96-6.73), 0.06 |
| Use of illicit drugs | 16.6 (-0.6-33.8), 0.06 | 2.86 (1.06-7.72), 0.04 |
| Homelessness | 16.1 (-1.2-33.3), 0.07 | 2.52 (0.95-6.71), 0.06 |
| Imprisonment | 14.6 (-3.00-32.2), 0.10 | 2.31 (0.86-6.20), 0.10 |
| Alcohol-related concerns | 18.0 (0.9-35.2), 0.04 | 2.57 (0.97-6.77), 0.06 |
| Smoking^ | - | - |
| HIV status^ | - | - |
| HBV vaccinated | 18.3 (0.1-36.4), 0.05 | 2.53 (0.96-6.66), 0.06 |
| Previous testing | 18.9 (1.4-36.3), 0.03 | 2.63 (0.99-6.98), 0.05 |
| Previous diagnosis | 18.1 (1.00-35.2), 0.04 | 2.53 (0.96-6.67), 0.06 |
| 'Known positive' at the time of recruitment | 17.3 (0.3-34.2), 0.05 | 2.72 (1.02-7.26), 0.05 |

*- binomial regression model did not converge, robust linear regression used to provide estimate, ^†-^ both age and age squared terms included, ^- smoking and HIV status collinear with the outcome. CI- confidence interval, HBV- hepatitis B virus, OR- odds ratio
